# Efficacy and Safety of Finerenone in Patients Aged 65 Years or Older with Diabetic Kidney Disease: Subgroup Analysis of the FINDKDLATAM Study

**DOI:** 10.3390/medicina62071317

**Published:** 2026-07-08

**Authors:** Jorge Rico Fontalvo, María Raad Sarabia, José C. De La Flor, Vicente Sánchez Polo, Jenniffer Benavides Garcia, Enrique Ramos Clason, Daniel Domínguez, Giovanny Mera Rebutti, Carlos Madrid Mancia, Rene Tabora López, Manuel Rocha Meza, Dany Tabora López, James J. Muñoz Zambrano, Eduardo Lorca Herrera, Eliana Dina-Batlle, Michael Cieza Terrones, Thyago Proença de Moraes, Tomas Rodríguez Yánez, Washington Osorio, Alyi Arellano Cabeza, Rodrigo Daza Arnedo

**Affiliations:** 1 Latin American Society of Nephrology and Hypertension (SLANH), Panama City 0801, Panama; jorgericof@yahoo.com (J.R.F.); visanpolo@gmail.com (V.S.P.); drdomingueznefrologia@gmail.com (D.D.); manuelrochameza@yahoo.com (M.R.M.); edulorcah@gmail.com (E.L.H.); dradina@me.com (E.D.-B.); michael.cieza@upch.pe (M.C.T.); thyago.moraes@pucpr.br (T.P.d.M.); dr.osoriow@yahoo.com (W.O.); rodrigoandres_2@hotmail.com (R.D.A.); 2 Colombian Association of Nephrology and Arterial Hypertension, Bogota 110221, Colombia; 3 Department of Nephrology, Faculty of Medicine, Simón Bolívar University of Barranquilla, Barranquilla 080001, Colombia; mariraad22@gmail.com; 4 Department of Nephrology, Hospital Central de la Defensa Gómez Ulla, 28047 Madrid, Spain; 5 Department of Medicine and Medical Specialties, Faculty of Medicine, Alcala University of Alcalá, 28805 Madrid, Spain; 6 Faculty of Medicine, Department of Nephrology, University of San Carlos of Guatemala (USAC), Guatemala City 01011, Guatemala; 7 Department of Epidemiology, El Rosario University, Health Science, Bogota 111221, Colombia; jennben89@gmail.com; 8 Postgraduate Medical and Surgical Research, GIBACUS Research Group, Sinú University EBZ, Cartagena Campus, Cartagena 130010, Colombia; drramosclason@gmail.com; 9 Faculty of Nursing, Health Services Administration Program, University of Cartagena, 13005 Cartagena, Colombia; 10 Department of Nephrology, Del Valle Hospital, Honduras, Rotating Internship at the Catholic University of Honduras, San Pedro Sula 21102, Honduras; drtaboralopezmi@gmail.com; 11 Department of Nephrology, Abel Gilbert Pontón Hospital of Guayaquil, Ministry of Public Health, O’Connors, Guayaquil 090104, Ecuador; gomerarebutti@gmail.com; 12 Department of Nephrology, Honduran Institute of Social Security, North Region, San Pedro Sula 21102, Honduras; drmadrid83@yahoo.com; 13 Department of Nephrology, Bendaña Hospital National Autonomous University of Honduras, San Pedro Sula 21102, Honduras; 14 Internal Medicine, Mario Catarino Rivas Hospital, San Pedro Sula 21102, Honduras; 15 Department of Nephrology, Del Valle Hospital, Catholic University of Honduras, San Pedro Sula 21102, Honduras; dannytabora29@gmail.com; 16 Department of Nephrology, Rafael Rodríguez Zambrano Hospital, Manta 130206, Ecuador; drjamesmunoz@hotmail.com; 17 Department of Internal Medicine East, Faculty of Medicine, University of Chile, Providencia, Santiago de Chile 8320000, Chile; 18 Department of Nephrology, Metropolitan Hospital of Santiago, Santiago de los Caballeros 51000, Dominican Republic; 19 Anglo American Clinic Nephrology Department, Alberto Hurtado Faculty of Medicine, Cayetano Heredia Peruvian University, Lima 15102, Peru; 20 Department of Nephrology, Pontifical Catholic University of Paraná, Curitiba 80060-000, Brazil; 21 Department of Internal Medicine, Faculty of Medicine, University of Cartagena, Cartagena 130010, Colombia; trodriguezy123@gmail.com; 22 Department of Nephrology, Armed Forces Specialty Hospital Number 1, Quito City 170403, Ecuador; 23 Department of Internal Medicine, University of Valle, Cali 760042, Colombia; alyi60428@gmail.com

**Keywords:** finerenone, diabetic kidney disease, older adults, albuminuria, Latin America

## Abstract

*Background and Objectives*: Diabetic kidney disease (DKD) is one of the leading causes of chronic kidney disease (CKD) worldwide. Individuals over 65 years of age with type 2 diabetes mellitus (T2DM) constitute a growing age group that is frequently underrepresented in randomized clinical trials. Finerenone has been shown to reduce renal progression and cardiovascular events in patients with albuminuric DKD. To evaluate the clinical and laboratory characteristics as well as the therapeutic response and safety profile of finerenone in patients over 65 years of age with DKD, based on data from the FINDKDLATAM observational study cohort. *Materials and Methods*: Subgroup analysis of a real-life, retrospective, multicenter observational study—the FINDKDLATAM cohort. For the present analysis, patients aged ≥ 65 years were stratified. Sociodemographic, clinical, paraclinical, and treatment variables and adverse events were evaluated. The descriptive analysis used medians with interquartile ranges (IQRs) for continuous variables of non-parametric distribution and absolute and relative frequencies for categorical variables. The comparison of baseline parameters and those at six months of follow-up was performed using non-parametric tests, with statistics. *Results*: Of the total of 347 patients included in the original study, 157 (45.2%) were ≥65 years of age. The median age was 72 years. The baseline profile showed a median glomerular filtration rate (eGFR) of 38.0 mL/min/1.73 m^2^ (IQR: 30.0–50.75), as well as elevated albuminuria (ACR 327.0 mg/g; IQR: 186.5–805.5). In addition, the patients had a high burden of comorbidities. 58.0% of patients received finerenone at a dose of 10 mg and 42.0% at a dose of 20 mg. After six months of follow-up, a significant reduction in the median ACR was observed, decreasing from 327.0 mg/g (IQR: 186.5–805.5) to 78.0 mg/g (IQR: 27.5–143.0), corresponding to a decrease of 76% (*p* < 0.0001). Regarding safety, hyperkalemia occurred in 20.3% of patients, although, in all cases, it was mild hyperkalemia (potassium between 5.1 and 5.5 mEq/L), with no serious episodes or treatment suspensions attributable to this event. *Conclusions*: In this cohort of Latin American adults over 65 years of age with albuminuric DKD, finerenone demonstrated good efficiency given the clinically significant reduction of albuminuria with stable renal function at six months of treatment, also demonstrating a safety profile, with a low risk of hyperkalemia.

## 1. Introduction

Diabetic kidney disease (DKD) has positioned itself as one of the main causes of chronic kidney disease (CKD) worldwide, being associated with a high risk of progression to end-stage renal disease and the occurrence of cardiovascular events [1]. As the population ages, the number of people over 65 years of age with type 2 diabetes mellitus (T2DM) and DKD increases, forming an increasingly large and relevant group of patients. However, this population segment has been underrepresented in recent therapeutic intervention studies, highlighting the need to analyze the effectiveness of treatment strategies in this group, evaluating both their clinical response and their safety profile [2].

In this population group, the coexistence of T2DM, CKD and established cardiovascular disease (CVD), which has been called cardiorenal metabolic syndrome (CRMS), represents a high-risk clinical scenario in which adverse outcomes mutually enhance each other [3]. Albuminuria has become one of the most robust prognostic markers, being an independent predictor of both renal progression and major cardiovascular events, including renal, cardiac and metabolic pathology [4,5].

Finerenone is a non-steroidal mineralocorticoid receptor antagonist (MRA) with a slightly different pharmacological profile compared to classic steroidal MRAs, such as spironolactone [6,7,8]. Its mechanism of action, centered on the selective inhibition of the mineralocorticoid receptor in the vasculature, heart and kidney, has demonstrated anti-inflammatory and anti-fibrotic effects that translate into both renal and cardiovascular benefits [8]. In the FIDELIO-DKD and FIGARO-DKD trials [9,10], the use of finerenone significantly reduced the risk of composite renal outcomes including sustained decline in estimated glomerular filtration rate (eGFR) and progression to end-stage renal failure and reduced the occurrence of major cardiovascular events in patients with CKD and type 2 diabetes.

One of the most consistently observed effects with finerenone is the early and sustained reduction of albuminuria, measured through the urinary albumin/creatinine ratio (UACR) [6,8]. In the FIDELIO-DKD and FIGARO-DKD trials, finerenone demonstrated significant reductions in albuminuria and favorable kidney outcomes in patients with CKD and type 2 diabetes [9,10]. More recently, prespecified secondary analyses from the FINEARTS-HF trial also evaluated kidney-related outcomes, including sustained eGFR decline, kidney failure, eGFR slope, and changes in urine albumin-to-creatinine ratio (UACR). In these analyses, finerenone was associated with a reduction in UACR and favorable kidney outcome trends in patients with heart failure [11]. These findings further support the potential renoprotective effects of finerenone across different cardiorenal populations.

The growing interest in finerenone has also been driven by the evolving concept of residual cardiorenal risk in patients with DKD. Despite the widespread use of renin-angiotensin system inhibitors and, more recently, SGLT2 inhibitors, a substantial proportion of patients continue to experience progressive kidney function decline and cardiovascular complications [4]. Persistent albuminuria has been recognized as one of the main indicators of this residual risk and remains an important therapeutic target [4]. In this context, finerenone has emerged as a complementary therapeutic option capable of addressing inflammatory and fibrotic pathways that are not fully controlled by conventional therapies, thereby expanding the therapeutic arsenal available for the management of diabetic kidney disease.

In numerous clinical trials and observational studies conducted in real-world settings, people over 65 years of age constitute a poorly represented group, although they have considerable clinical and epidemiological interest. The aging process is associated with an increased prevalence of CKD, greater cumulative exposure to factors that negatively affect kidney function, and increased susceptibility to adverse events related to certain drug substances.

In addition to the progressive decline in kidney function, older adults with DKD frequently present with multimorbidity, polypharmacy, frailty, and functional limitations that may influence treatment selection and clinical outcomes. These factors often complicate the implementation of evidence-based therapies and increase concerns regarding adverse events, particularly in patients with advanced CKD. Consequently, there is a growing interest in identifying therapeutic strategies capable of providing renal and cardiovascular protection while maintaining an acceptable safety profile in elderly populations. Real-world evidence is particularly valuable in this setting because it reflects the characteristics and complexity of patients commonly encountered in daily clinical practice, many of whom would not meet the eligibility criteria of randomized clinical trials. This is especially relevant for older adults, who remain underrepresented in clinical research despite bearing a substantial burden of DKD and related cardiovascular complications, highlighting the need for data that better inform treatment decisions in this vulnerable population.

The specific characterization of albuminuria reduction with the use of finerenone in the population over 65 years of age remains insufficiently explored in focused analyses. The purpose of this work is to evaluate the magnitude and consistency of the reduction of albuminuria with the use of finerenone in patients aged 65 years or older, based on the evidence available in the FINDKDLATAM observational study [12], in order to inform the behavior of this drug substance in this population group and provide solid evidence for clinical decision-making in this age group of increasing epidemiological relevance.

## 2. Materials and Methods

### 2.1. Type of Study

A subgroup analysis of the FINDKDLATAM cohort (12) was performed, an observational, multicenter, real-life data-based study designed to evaluate the effectiveness and safety of finerenone in Latin American patients with CKD associated with T2DM. The original cohort included patients treated in seven Latin American countries, for whom finerenone was prescribed as part of routine clinical practice in combination with other standard therapies. For this manuscript, only patients aged 65 years or older at the time of inclusion were selected, with the purpose of characterizing the therapeutic response and safety profile in the older adult’s population.

### 2.2. Study Population

The eligible population consisted of adult patients diagnosed with type 2 diabetes and chronic kidney disease, albuminuria defined by a urinary albumin/creatinine ratio (ACR) greater than 30 mg/g, relevant clinical history of cardiovascular risk, and pharmacological treatment for diabetes-associated chronic kidney disease who participated in the FINDKDLATAM cohort. For this analysis, only patients aged ≥65 years were included. Complete baseline and 6-month follow-up data were available for all 157 eligible patients, no patients were excluded because of missing follow-up information, and no losses to follow-up occurred during the study period for this age subgroup.

### 2.3. Exposure and Intervention

The exposure evaluated was the use of finerenone at a daily dose of 10 mg or 20 mg, prescribed by the treating physician in accordance with usual clinical practice, renal function, serum potassium levels and current clinical guideline recommendations. No randomization or experimental intervention was performed. Concomitant management, including renin–angiotensin–aldosterone system inhibitors (RAASis), sodium–glucose cotransporter type 2 inhibitors (SGLT2is), GLP-1 receptor agonists (aGLP-1), and other cardiometabolic drugs, was recorded as part of the standard treatment received by each patient.

### 2.4. Variables and Outcomes

Sociodemographic, clinical, paraclinical, and therapeutic variables were collected. Baseline variables included age, sex, body mass index (BMI), history of high blood pressure (HBP), CVD, acute myocardial infarction (AMI), smoking, glycated hemoglobin levels (HbA1%), eGFR, ACR, and serum potassium. The effectiveness outcomes were the change in ACR and eGFR between baseline and six months of follow-up. The antiproteinuric response was classified according to the percentage reduction in the ACR into three categories: >50% reduction, reduction between 30% and 50%, and absence of significant response. The primary safety outcome was the occurrence of hyperkalemia during follow-up, classified according to available potassium values.

To better characterize the clinical profile of the study population, baseline demographic and comorbidity data were analyzed in conjunction with laboratory and treatment variables. Follow-up assessments were performed according to routine clinical practice at each participating center, reflecting a real-world care model rather than a protocol-driven intervention. Laboratory measurements were obtained from local accredited laboratories, and the values recorded closest to baseline and six months after finerenone initiation were used for analysis. This approach allowed the evaluation of treatment effectiveness under conditions representative of everyday nephrology practice across different healthcare systems in Latin America.

Cardiovascular history was assessed based on the information available in the medical records and included the presence of established cardiovascular disease and previous acute myocardial infarction. Established cardiovascular disease was defined as documented heart failure, cerebrovascular disease, peripheral arterial disease. Previous acute myocardial infarction was recorded separately as an independent baseline variable according to the original FINDKDLATAM database structure. These variables were recorded as baseline comorbidities because of their recognized association with adverse renal and cardiovascular outcomes in patients with DKD. Likewise, serum potassium values obtained during routine follow-up were reviewed to identify episodes of hyperkalemia after finerenone initiation. Because of the retrospective design of the study, detailed information regarding previous episodes of hyperkalemia before treatment initiation, their severity, duration, precipitating factors, or specific management strategies was not consistently available across all participating centers and therefore could not be systematically analyzed. Consequently, the safety assessment focused on hyperkalemia events documented during the follow-up period after finerenone prescription.

### 2.5. Statistical Analysis

Statistical analysis was performed using a descriptive and comparative approach for paired data. Categorical variables are presented as absolute and relative frequencies. Continuous variables are described using median and interquartile range (IQR), according to the non-parametric distribution observed in the original cohort. The comparison of baseline parameters and those at six months was performed using the Wilcoxon signed-rank test for paired samples; therefore, confidence intervals for paired differences were not routinely estimated. For the comparison of categorical variables, when appropriate, the χ^2^ test or Fisher’s exact test was used depending on the expected frequency distribution. A *p*-value < 0.05 was considered statistically significant.

Given the observational nature of the study, the analyses were primarily exploratory and descriptive. No imputation procedures were required because complete baseline and follow-up information was available for all patients included in this subgroup analysis. The magnitude of changes in renal and metabolic parameters was evaluated through paired comparisons, allowing each participant to serve as their own control over time. This methodological approach minimizes interindividual variability and provides a pragmatic assessment of treatment performance under routine clinical conditions.

No formal adjustment for multiple comparisons was performed because the analyses were exploratory in nature and aimed primarily to describe changes in clinical and laboratory parameters over time in this real-world cohort. Therefore, *p*-values should be interpreted as descriptive measures of statistical association.

Statistical analysis was performed using the SPSS statistical program version 30.

### 2.6. Ethics Committee

The FINDKDLATAM study was approved by the ethics committee of the Faculty of Medical Sciences of the University of San Carlos of Guatemala and was developed in accordance with the principles of the Declaration of Helsinki. Participants in the original study were informed about the nature of the study and signed informed consent. For this subgroup analysis, data derived from the original cohort were used, without additional interventions and maintaining the confidentiality of clinical information.

## 3. Results

The original study included a total of 347 patients. This subgroup analysis included 157 patients aged 65 years or older. The median age of this population was 72 years (IQR 68–77), females represented 38.9% of the population (n = 61) while 61.1% were male (n = 96). Regarding BMI, the median was 28.00 (IQR 26.00–31.48).

Regarding the behavior of the renal variables, the median baseline eGFR was 38.00 (IQR 30.00–50.75), and the median baseline UACR was 327.00 mg/g (IQR 186.5–805.50). Baseline potassium levels remained stable with a median of 4.40 mEq/L (IQR 4.10–4.70). Regarding glycemic control, the baseline HbA1c had a median of 7.35% (IQR 6.6–8.0%). Regarding clinical history, 100% of patients over 65 years of age had HBP, 49% had CVD, 83.4% had exposure to tobacco, and 75.8% had a history of acute myocardial infarction (AMI) (Table 1).

When comparing baseline values with those recorded at 6 months of follow-up, we observed a significant improvement in ACR levels with a substantial reduction, going from a median of 327.00 mg/g (IQR: 186.50–805.50) to 78.00 mg/g (IQR: 27.50–143.00; *p* < 0.0001), which suggests a relevant decrease in albuminuria during follow-up (76% reduction). The eGFR remained stable during the evaluated period, with no statistically significant differences between the baseline value and the control at 6 months. Regarding hyperkalemia as a safety outcome, it occurred in 20.3% of the population; however, these cases were at levels of mild hyperkalemia (5.1–5.5 mEq/L). No patient required finerenone dose reduction or discontinuation, potassium-binding agents, emergency medical interventions, or specific dietary potassium restriction attributable to hyperkalemia (Table 2).

Additional analysis of laboratory and clinical parameters demonstrated an overall favorable evolution during follow-up. Along with the marked reduction in albuminuria, glycemic control showed a modest but statistically significant improvement, reflected by the decrease in median HbA1c levels. Likewise, both systolic and diastolic blood pressure values were lower at six months compared with baseline measurements. At six months of follow-up, additional changes were observed in several clinical and laboratory parameters. Median HbA1c decreased from 7.35% (IQR 6.60–8.00) at baseline to 7.00% (IQR 6.50–7.70) at follow-up (*p* < 0.0001). Median systolic blood pressure decreased from 145 mmHg (IQR 134–158) to 130 mmHg (IQR 120–140), while median diastolic blood pressure decreased from 80 mmHg (IQR 70–82) to 72 mmHg (IQR 70–80) (both *p* < 0.0001).

**Figure 1:**
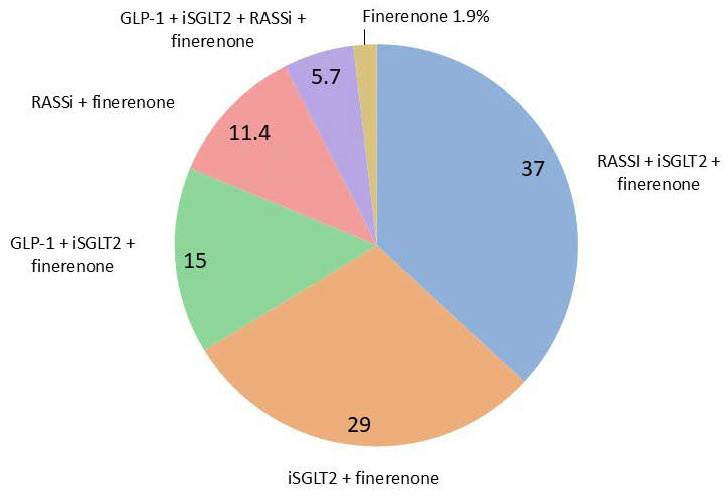
Therapeutic group distribution in patients over 65 years of age. Abbreviations: SGLT2i: Sodium–Glucose Cotransporter 2 inhibitors; RAASi: Renin–Angiotensin–Aldosterone System inhibitor; GLP-1: Glucagon-Like Peptide-1.

Regarding the therapeutic group, the distribution of therapy in patients over 65 years of age was distributed as follows: RAASi + SGLT2i + finerenone 37%, SGLT2i + finerenone 29%, GLP-1 + SGLT2i + finerenone 15%, RAASi + finerenone 11.4%, aGLP-1 + SGLT2i + RAASi + finerenone 5.7%, finerenone monotherapy 1.9%. In terms of albuminuria response, 128 patients (81.53%) achieved a reduction greater than 50% in ACR, 21 patients (13.38%) showed a reduction between 30% and 50%, and 8 patients (5.10%) did not meet the predefined response criteria (Figure 1).

**Table 1:** Baseline clinical and laboratory characteristics of patients over 65 years of age.

Characteristics	Value
**Demography**	
Age [years], Median [IQR]	72.00 [68.00–77.00]
Female Sex, n (%)	61 (38.9%)
**Baseline Clinical Parameters**	
BMI [kg/m^2^], Median [IQR]	28.00 [26.00–31.48]
eGFR [mL/min/1.73 m^2^], Median [IQR]	38.00 [30.00–50.75]
ACR [mg/g], Median [IQR]	327.00 [186.50–805.50]
HbAlc [%], Median [IQR]	7.35 [6.60–8.00]
SBP [mmHg], Median [IQR]	145.00 [134.00–158.00]
DBP [mmHg], Median [IQR]	80.00 [70.00–82.00]
Potassium [mEq/L], Median [IQR]	4.40 [4.10–4.70]
**Medical History, n (%)**	
High Blood Pressure	157 (100.0%)
Cardiovascular Disease	77 (49.0%)
Smoking	131 (83.4%)
Acute Myocardial Infarction	119 (75.8%)
Obesity	103 (65.6%)
**Previous Pharmacological Treatment, n (%)**	
SGLT2i	139 (88.5%)
RAASi	92 (58.6%)
GLP-1	29 (18.5%)
**Finerenone Dose, n (%)**	
10 mg	91 (58.0%)
20 mg	66 (42.0%)

Abbreviations: [IQR]: interquartile range, n: patients; %: percentage; BMI: body mass index; eGFR: glomerular filtrate rate; ACR: Albumin-to-creatinine ratio; HbA1c: hemoglobin A1c; SBP: systolic blood pressure; DBP: diastolic blood pressure; SGLT2i: Sodium–Glucose Cotransporter 2 inhibitors; RAASi: Renin–Angiotensin–Aldosterone System inhibitor; GLP-1: Glucagon-Like Peptide-1.

**Table 2:** Comparison of baseline and final parameters.

Characteristics	Baseline Value	Value at 6 Months	*p*-Value
**Clinical Parameters**			
eGFR (mL/min/1.73 m^2^), Median [IQR]	38.00 [30.00–50.75]	40.00 [28.25–55.00]	0.7231
ACR (mg/g), Median [IQR]	327.00 [186.50–805.50]	78.00 [27.50–143.00]	<0.0001
HbA1c (%), Median [IQR]	7.35 [6.60–8.00]	7.00 [6.50–7.70]	<0.0001
SBP (mmHg), Median [IQR]	145.00 [134.00–158.00]	130.00 [120.00–140.00]	<0.0001
DBP (mmHg), Median [IQR]	80.00 [70.00–82.00]	72.00 [70.00–80.00]	<0.0001
Potassium (mEq/L), Median [IQR]	4.40 [4.10–4.70]	4.80 [4.40–5.00]	<0.0001

Abbreviations: [IQR]: interquartile range, eGFR: glomerular filtrate rate; ACR: Albumin-to-creatinine ratio; HbA1c: hemoglobin A1c; SBP: systolic blood pressure; DBP: diastolic blood pressure.

## 4. Discussion

The present observational study corresponds to a subgroup analysis of the real-life FINDKDLATAM study cohort (12) which included 347 patients with T2DM and CKD, of whom 45.2% were over 65 years old, an age group usually underrepresented in clinical trials but which constitutes a growing proportion in real clinical practice.

In this cohort, patients had a very high cardio-renal risk profile, characterized by CKD stage 3b (median eGFR of 38 mL/min/1.73 m^2^), elevated baseline albuminuria levels (median ACR of 327.0 mg/g) and a high burden of cardiovascular comorbidity, with 49% established CVD and 100% with HBP. Despite this risk profile, a remarkably high rate of therapeutic response was observed, with 81.5% of patients achieving a reduction of more than 50% in the ACR, suggesting an anti-proteinuric effect in this age group.

During the 6-month follow-up, we observed a significant decrease in the ACR, approximately 76%; only 5.1% of patients had a non-significant response in terms of albuminuria reduction. In FIDELIO-DKD (10), the reduction in the UACR was on the order of 30% in the first months, while in FIGARO-DKD (9) and combined analyses of the FIDELITY program, the effects were consistent but of lower average magnitude. Similarly, the FINDKDLATAM study in the global population reported reductions of approximately 70–76% under real clinical practice conditions [12]. Although the magnitude of albuminuria reduction observed in this cohort differs from that reported in pivotal randomized trials, direct comparisons should be avoided because of differences in study design, baseline risk profile, concomitant therapies, and the absence of a control group in the present analysis. Therefore, these results should be interpreted as supportive real-world evidence of albuminuria reduction in patients aged 65 years or older.

An important characteristic of our cohort was the exceptionally high burden of cardiovascular disease observed in an older adult with diabetic kidney disease. The median age was 72 years, and more than three-quarters of patients had a prior history of acute myocardial infarction, reflecting a population at extremely high cardiorenal risk. This prevalence appears higher than that reported in most randomized clinical trials and observational cohorts of patients with diabetic kidney disease. The advanced age of the population, together with the high prevalence of cardiovascular comorbidities, suggests that this cohort represents a particularly vulnerable subgroup frequently encountered in practice but often underrepresented in clinical trials.

The objective of this study was not to compare the efficacy and safety of finerenone between different age groups, unlike what was done by Bansal S. et al. [13] who carried out a comparative analysis in the FIDELITY cohort. This analysis demonstrated that the cardiovascular and renal protection provided by finerenone is not influenced by age, and that renal events decreased with the use of finerenone regardless of age group, suggesting that age is not a modulating factor of the renal protection of this drug. The lack of statistical significance in the group over 75 years old could be attributed to the small number of events and the small representation of patients in that subgroup compared to the other age groups analyzed.

The question about the efficacy of finerenone in older patients is very pertinent since the activity of the enzyme 11B hydroxysteroid dehydrogenase type 2, which protects against the overactivity of the mineralocorticoid receptor (MR) by cortisol, is downregulated with advanced age, so, in older patients, there could be overactivity of the MR by cortisol, so theoretically they would be patients with a greater probability of benefiting from blocking the MR [14,15]. Similarly, a lower activity of the enzyme 11 B hydroxysteroid dehydrogenase has been identified in men with HBP [16], which could impact the activity of finerenone in this population. Our study did not assess potential differences in efficacy and safety in terms of biological sex.

Other therapies considered as a standard of care in patients with T2DM and cardiovascular risk, such as SGLT2 inhibitors [7,17], have also shown that their benefit is independent of the patient’s age and also impact renal outcomes and control of serum glucose levels [18,19]. In fact, a trend towards greater benefits has been observed in more frail patients [20], these findings are very plausible given that these patients had a higher absolute risk at baseline.

In the FOUNTAIN program cohort in the United States, the average age of patients treated with finerenone was 70.3 years, very similar to that observed in our population (median of 72 years), suggesting adequate comparability in terms of aging and baseline risk burden. However, there are relevant differences in the magnitude and dynamics of the response: while in FOUNTAIN the reduction in the ACR is documented progressively from the fourth month and is maintained until 12 months, in our Latin American cohort, this effect was earlier and more pronounced, with a decrease of close to 76% already at six months. This discrepancy could reflect differences in the baseline albuminuria profile, the intensity of concomitant treatment (particularly combined use with SGLT2i and renin-angiotensin system blockade) and the characteristics of clinical practice in each setting. In addition, given the observational before-and-after design and the markedly elevated baseline UACR values, part of the observed reduction may have been influenced by regression to the mean and the natural biological variability of albuminuria measurements. Nevertheless, under real-life conditions in Latin America, the present findings are consistent with an antiproteinuric effect of finerenone in patients over 65 years of age, while the magnitude of this reduction should be interpreted cautiously given the observational before-and-after design and the potential contribution of regression to the mean [21].

One particularly relevant aspect is the stability of the eGFR. Unlike what has been described with other nephroprotective drugs, where an initial drop in eGFR may be observed, in this cohort, no significant changes were documented at six months. This finding is consistent with what has been reported in observational studies and subgroup analyses, where the effect of finerenone is characterized more by slowing the slope of decline than by acute changes in renal function [22].

Regarding safety, the incidence of hyperkalemia was mostly mild and did not lead to the discontinuation of treatment. This pattern is consistent with that observed in FIDELIO-DKD (10), where hyperkalemia was more frequent with finerenone, but rarely resulted in clinically serious events. In older adults, this point is especially relevant, since concern about electrolyte imbalances often limits the use of MR antagonists. However, in this study, we found an incidence of hyperkalemia of 20.3%, slightly higher than the general cohort of FINDKDLATAM, which could be influenced by age, since being over 65 years old acts as an independent risk factor for the development of hyperkalemia [23]. The data presented in our study suggest that, under adequate monitoring, the safety profile is manageable in patients over 65 years of age, even in patients with compromised renal function.

However, these findings should be interpreted considering some limitations. The observational design introduces potential selection bias and residual confounding, particularly regarding the treatment indication and the intensity of concomitant therapies. In addition, inclusion in the present subgroup analysis required the availability of complete baseline and six-month follow-up data. Although no losses to follow-up occurred among the patients included in this subgroup, this requirement may have excluded individuals who discontinued treatment, transferred care, or lacked complete follow-up information. Consequently, the efficacy and safety estimates reported herein should be interpreted with this potential source of selection bias in mind. Furthermore, the absence of a control group limits the ability to establish causal relationships, making it impossible to determine what proportion of the observed reduction in albuminuria is attributable solely to finerenone use and not to other factors, such as the natural variability of albuminuria levels or the intensification of concomitant treatments. Another important limitation is the potential influence of regression to the mean. Because baseline UACR values were markedly elevated and showed substantial variability, part of the observed reduction in albuminuria may reflect natural fluctuations in repeated measurements rather than the treatment effect alone. Therefore, although the magnitude of albuminuria reduction is consistent with the known antiproteinuric effects of finerenone, the contribution of regression to the mean cannot be completely excluded in the absence of a control group. The relatively short follow-up period precludes the evaluation of critical outcomes such as progression to renal replacement therapy or major cardiovascular events. In addition, the cohort represents a specific Latin American setting, which may limit the generalizability of the results to other contexts.

Despite these limitations, the study provides relevant information for clinical practice. Our study has many strengths; we highlight that it is the largest published real-life cohort of Latin American population formulated with finerenone across 7 different countries in the region. In a scenario where the population with diabetic kidney disease is aging rapidly, having specific data on those over 65 years of age is essential. The magnitude of the observed reduction in albuminuria, along with the stability of renal function and an acceptable safety profile, positions finerenone as a solid therapeutic tool in this age group.

## 5. Conclusions

In this cohort of patients over 65 years of age with DKD, finerenone is associated with a significant reduction in albuminuria, with more than 80% of patients achieving a significant response (>50% reduction in UACR), in addition to being a safe medication with a good safety and tolerability profile. These findings support the use of finerenone as an effective and safe therapeutic strategy within a multidisciplinary and combined approach, especially in geriatric populations frequently underrepresented in clinical trials. Prospective and long-term follow-up studies are needed to confirm its impact on hard renal and cardiovascular outcomes in this age group.

## Data Availability

No new data were created or analyzed in this study. The data used to support the findings of this study are available from the principal author on request (contact Jorge Rico Fontalvo, email: jorgericof@yahoo.com).
